# Reduced Serum Vitamin D Levels Are Associated with Insulin Resistance in Patients with Obstructive Sleep Apnea Syndrome

**DOI:** 10.3390/medicina55050174

**Published:** 2019-05-20

**Authors:** Kostas Archontogeorgis, Nikolaos Papanas, Evangelos C. Rizos, Evangelia Nena, Athanasios Zissimopoulos, Christina Tsigalou, Athanasios Voulgaris, Dimitri P. Mikhailidis, Moses S. Elisaf, Marios E. Froudarakis, Paschalis Steiropoulos

**Affiliations:** 1 MSc Programme in Sleep Medicine, Medical School, Democritus University of Thrace, 68100 Alexandroupolis, Greece; k.archontogeorgis@yahoo.it; 2 Diabetes Centre, Second Department of Internal Medicine, Medical School, Democritus University of Thrace, 68100 Alexandroupolis, Greece; papanasnikos@yahoo.gr; 3 Department of Internal Medicine, School of Medicine, University of Ioannina, 45110 Ioannina, Greece; vagrizos@gmail.com (E.C.R.); melisaf54@gmail.com (M.S.E.); 4 Laboratory of Hygiene and Environmental Protection, Medical School, Democritus University of Thrace, 68100 Alexandroupolis, Greece; enena@med.duth.gr; 5 Laboratory of Nuclear Medicine, Medical School, Democritus University of Thrace, 68100 Alexandroupolis, Greece; azisimop@med.duth.gr; 6 Laboratory of Microbiology, Medical School, Democritus University of Thrace, 68100 Alexandroupolis, Greece; xtsigalou@yahoo.gr; 7 Department of Pneumonology, Medical School, Democritus University of Thrace, 68100 Alexandroupolis, Greece; thanasisvoul@hotmail.com (A.V.); mfroud@med.duth.gr (M.E.F.); 8 Department of Clinical Biochemistry, Royal Free Hospital Campus, University College London Medical School, University College London (UCL), NW3 2QG London, UK; mikhailidis@aol.com

**Keywords:** insulin resistance, obesity, obstructive sleep apnea syndrome, vitamin D

## Abstract

*Background and objectives*: Obstructive sleep apnea syndrome (OSAS) is associated with cardiovascular and metabolic risk factors, such as insulin resistance. Furthermore, OSAS has been associated with decreased levels of vitamin D (Vit D). The aim of the study was to assess the association between Vit D levels (expressed as 25(OH)D serum levels) and insulin resistance in patients with OSAS. *Materials and Methods:* Serum 25(OH)D levels were measured in consecutive subjects who had undergone polysomnography and pulmonary function testing. OSAS patients were divided into those with (homeostatic model assessment [HOMA-IR] ≥ 2) and without insulin resistance (HOMA-IR < 2). *Results:* Overall, 92 patients (81 males) were included in the study. OSAS patients with insulin resistance significantly differed from those without insulin resistance in terms of the body-mass index (BMI) (36.3 ± 5.8 compared to 32 ± 5.6 kg/m^2^, respectively, *p* = 0.001), apnoea-hypopnoea index (AHI) (57.4 ± 28.9 compared to 40.9 ± 27.9 events/h, respectively, *p* = 0.009) and indices of hypoxia during sleep. Patients with OSAS and insulin resistance had lower levels of serum 25 (OH) D compared with OSAS but without insulin resistance (19.3 ± 11.5 *vs* 26.7 ± 12.2 ng/mL, respectively, *p* = 0.005). Regression analysis demonstrated a negative association of 25(OH)D levels (β = −0.048, odds ratio [OR]: 0.953, 95% confidence interval [CI]: 0.913–0.995, *p* = 0.030) and a positive association of BMI (β = 0.110, OR: 1.116, 95% CI: 1.007–1.237, *p* = 0.036) with insulin resistance. *Conclusions:* Vit D insufficiency was significantly more frequent among OSAS patients with insulin resistance. Both low 25(OH)D levels and high BMI were associated with the risk of insulin resistance in this population.

## 1. Introduction

The hallmark of obstructive sleep apnea syndrome (OSAS) is repetitive episodes of partial (hypopnea) or complete (apnea) occlusion of the upper airway during sleep, which lead to oxygen desaturation and sleep fragmentation [1]. OSAS is now highly prevalent in the Western countries [2]. Its main risk factors are older age, male sex, obesity, family history, menopause, craniofacial abnormalities, cigarette smoking and alcohol use [3]. The consequences of OSAS include excessive daytime sleepiness, cognitive dysfunction, and impoverished quality of life [4]. There is evidence linking OSAS with cardiovascular risk factors, such as arterial hypertension, coronary heart disease, cerebrovascular disease and overall mortality [5]. In addition, OSAS has been associated with insulin resistance [6].

Vitamin D (Vit D), a fat-soluble vitamin, plays a pivotal role in bone and mineral metabolism [7]. It is produced in the skin after exposure to solar radiation or acquired from oral intake (natural foods, fortified foods, and supplements) [7]. Serum 25-hydroxyvitamin D [25(OH)D] is recommended as the marker of choice for the evaluation of the Vit D status [8]. Vit D insufficiency is implicated in several pulmonary diseases, such as viral and bacterial respiratory infections, asthma, chronic obstructive pulmonary disease and cancer [9]. In addition, accumulating data point to an association between serum Vit D levels and presence of OSAS [10,11,12]. Vit D has been shown to be associated with glucose homeostasis, affecting insulin sensitivity as well as the β-cell function [13]. Its levels are often decreased in patients with insulin resistance (IR) or diabetes mellitus (DM) [13]. Low levels of Vit D may also be associated with increased risk of diabetes mellitus and cardiovascular disease [14,15,16,17].

The aim of this study was to examine the potential association between Vit D levels and insulin resistance in patients with OSAS.

## 2. Materials and Methods

### 2.1. Patients

The study included consecutive patients referred to our sleep laboratory during a six-month period (May and November 2017) for suspected OSAS. All investigations were carried out in accordance with the Helsinki Declaration of Human Rights [18]. The study was approved by the institutional ethics committee (Ethical approval number 54/19.12.2014) and patients gave their informed consent.

Exclusion criteria were: known DM; central sleep apnea; corticosteroid and/or hormonal replacement therapy; treatment with Vit D and calcium supplementation; conditions known to interfere with Vit D, calcium and phosphorus metabolism; severe heart failure; inflammatory diseases; cancer; chronic pulmonary, liver or renal disease; and osteoporosis.

Previous medical history, current medication use, with emphasis on Vit D supplements and glucose lowering drugs, tobacco smoking and alcohol consumption were recorded. Diagnosis of diabetes mellitus was based on the medical history or on levels of fasting plasma glucose ≥126 mg/dL [19]. Height, weight, as well as neck circumference, hip circumference, waist circumference and waist/hip ratio were measured using standardized techniques. The body-mass index (BMI) was calculated according to the formula: Weight (kg)/height^2^ (m).

Sleepiness was assessed using the validated Greek version of the Epworth Sleepiness Scale (ESS), a self-administered eight-item questionnaire evaluating the possibility of dozing in a variety of situations (maximum score: 24; score >10 indicative of excessive daytime sleepiness) [20].

Pulmonary function testing, arterial blood gases’ analysis and a 12-lead electrocardiogram were also performed before polysomnography (PSG).

### 2.2. Polysomnography

Participants underwent full in-laboratory PSG, attended by an experienced sleep technician, from 22:00 to 06:00 hours and variables were recorded on a computer system (Alice^®^ 4, Philips Respironics, Murrysville, PA, USA). A standard montage of electroencephalogram, electrooculogram, electromyogram and electrocardiogram signals was used. Arterial oxygen saturation was recorded by a digital pulse oximeter and airflow was detected using combined oronasal thermistors. The thoracic cage and abdominal motion were also recorded using piezoelectric bands placed around the chest and abdomen. Respiratory events and electroencephalogram recordings were manually scored according to the standard criteria [21]. Apnoea was defined as a ≥90% of reduction in airflow for at least 10 sec [21]. Hypopnea was defined as a ≥30% reduction in airflow for at least 10 sec in combination with oxyhaemoglobin desaturation of at least 3% or an arousal registered by the electroencephalogram [21]. The apnoea-hypopnea index (AHI) was calculated as the average number of apnoeas and hypopneas/h of PSG-recorded sleep time [21]. OSAS was defined as AHI ≥5/h accompanied by related symptoms [22].

### 2.3. Blood Samples and Measurements

Blood samples for insulin and Vit D levels (assessed as 25(OH)D) and other biochemical examinations were drawn the day after PSG. All blood samples were taken after fasting for at least 8 h. Blood samples were centrifuged (3000 rpm for 10 min) and the serum obtained was kept frozen at −80 °C for future analysis. Serum 25(OH)D was measured using a commercial radioimmunoassay kit (DiaSorin, Stillwater, MN, USA). Insulin levels were measured using an electrochemiluminescence immunoassay (ECLIA) on a Cobas immunoassay analyzer. IR was assessed by the Homeostasis Model Assessment of Insulin Resistance (HOMA-IR = fasting insulin (mU/L) × fasting glucose (mg/dL)/405) [23]. A HOMA-IR threshold >2 was used to determine insulin resistance [24,25].

### 2.4. Statistical Analysis

All analyses were performed using the IBM Statistical Package for Social Sciences version 17.0 (SPSS Inc, Chicago, Il, 2008,). Continuous variables were tested for normality of distribution by the Kolmogorov–Smirnov test. Normally distributed quantitative data were expressed as the mean ± standard deviation and those with skewed distribution as the median (25th–75th percentile). Comparisons were carried out with the Student t-test (normal distribution) or the Mann–Whitney test (skewed distribution). The chi-square test was used for comparison of percentages between groups. Correlations were analyzed with Pearson’s correlation coefficient. Associations between variables on the logistic regression analysis with a two-tailed *p* < 0.05 were entered into multivariate models to determine the independent correlations with insulin resistance. A two-tailed *p* < 0.05 was considered significant.

## 3. Results

Overall, 92 OSAS patients (81 males and 11 females; mean age of 50 ± 11.7 years) were included. In the entire OSAS population, mean serum Vit D levels were 22.1 ± 12.2 ng/mL and HOMA-IR was 3.5 (1.7–7.7). There was no association between Vit D levels and HOMA-IR (*p* > 0.05).

OSAS patients were divided according to their HOMA-IR into group A without IR, (HOMA-IR < 2), (34 patients: Twenty-nine males and five females) and group B with IR, (HOMA-IR ≥ 2), (58 patients: fifty-two males and six females). BMI was significantly higher in group B than in group A (36.3 ± 5.8 *vs* 32 ± 5.6 kg/m^2^ respectively, *p* = 0.001). Anthropometric and demographic characteristics of both groups are presented in Table 1.

Patients in group B exhibited increased AHI compared with group A (57.4 ± 28.9 compared to 40.9 ± 27.9 events/h, respectively, *p* = 0.009) and had worse indices of hypoxia during sleep. Sleep characteristics are presented in Table 2.

The two groups differed significantly in terms of oxygen partial pressure, glucose and triglycerides levels. Serum 25(OH)D levels were decreased in group B (19.3 ± 11.5 compared to 26.7 ± 12.2 ng/mL, respectively, *p* = 0.005). Laboratory results are presented in Table 3.

In order to establish principal predictors of IR among OSAS patients, the regression analysis was applied considering BMI, serum 25(OH)D, AHI and indices of hypoxia during sleep as independent variables. It was demonstrated that BMI (β = 0.110, OR: 1.116, 95% CI: 1.007–1.237, *p* = 0.036) and 25(OH)D serum levels (β = −0.048, OR: 0.953, 95% CI: 0.913−0.995, *p* = 0.030), but not their interaction (*p* = 0.084), were the main predictors of IR.

## 4. Discussion

This study has shown an association between reduced serum 25(OH)D levels and IR in OSAS patients. Moreover, BMI and levels of serum 25(OH)D were found to be independent predictors of IR in these patients. These two variables did not interact however, suggesting that the association between serum 25(OH)D levels and IR was not significantly mediated by obesity in our study.

The association between Vit D levels and IR has long been disputed. In a cross-sectional study assessing 446 subjects with metabolic syndrome, 25(OH)D concentrations were not associated with insulin action or secretion, as evaluated by both the HOMA-IR and intravenous glucose tolerance test, after adjustment for BMI and other covariates [26]. In addition, Vit D supplementation did not improve glycaemia or insulin resistance in patients with DM, normal fasting glucose or impaired glucose tolerance [27,28].

Conversely, in a four-year follow-up study, decreased 25(OH)D levels were significantly associated with the risk of developing prediabetes (OR: 3.01, 95% CI: 1.50–6.06, *p* = 0.002) and type2 DM (OR: 5.61, 95% CI: 1.73–18.27, *p* = 0.004) [29]. Moreover, low baseline 25(OH)D levels were an independent predictor of increased IR [29]. Additionally, recent meta-analyses have shown that Vit D supplementation reduces insulin resistance in selected patient series [30,31]. Taken together, the data have not yet clearly shown whether low Vit D levels are the result of or the cause of IR.

In our study, Vit D levels were lower in OSAS patients with IR. Our results concur with those of previous studies [32]. Bozkurt et al. [33] have reported lower 25(OH)D levels in OSAS subjects with IR (*p* = 0.004). 25(OH)D levels decreased as patients with OSAS progressed from IR to prediabetes and DM (*p* = 0.02) [33]. Similarly, in another study, decreased risk of DM (*p* = 0.038) and metabolic syndrome (*p* < 0.001) was observed with increasing Vit D levels in OSAS patients, after adjustment for age, sex and seasonality [34].

Our results have identified that low Vit D levels and high BMI are associated with IR in OSAS patients. Obesity, one of the main characteristics of OSAS patients, is a well-known risk factor of IR [35]. Obesity may also adversely affect Vit D levels through various mechanisms. These include lower dietary intake or less sunlight exposure, as well as Vit D sequestration in fat tissue and reduced bioavailability [36,37,38]. Adipose tissue presents lower expression of the enzymes responsible for Vit D activation and a propensity towards increased catabolism [39]. Furthermore, dilution of ingested or cutaneously synthesized Vit D in the large fat mass of obese patients may reduce Vit D levels [40].

Thus, considering the contradicting data regarding the relationship between Vit D insufficiency and IR, as well as its association with obesity, it becomes difficult to establish whether low serum Vit D levels represent an independent risk factor for IR or this particular effect is mediated by obesity.

Of practical relevance, it has recently been confirmed that OSAS is frequently associated with several vascular risk factors, and consequently with a greater risk of vascular events, and so it is likely that some of these patients will need statins [41,42,43]. In turn, there is evidence that Vit D deficiency is associated with a higher incidence of statin-related myopathy [44]. This may affect adherence to treatment [45] and highlights a further consequence of Vit D deficiency in these patients.

This study has some limitations. The first is the small patient number. Larger studies are necessary to assess better the association of Vit D levels and IR in OSAS. Secondly, in our study included were middle-aged patients, and so our results should be extrapolated with caution to older OSAS individuals. Moreover, we lacked data on skin pigmentation and dietary habits. However, the study was conducted in a relatively short time (six-month interval) among Caucasian inhabitants of the same area, with similar dietary habits, and assessed with the gold standard assay of Vit D during a period characterized of poor sunlight exposure and without any protective sunscreen that could interfere with Vit D measurements. Finally, polymorphisms of Vit D receptor and Vit D binding protein genes were not investigated in this study. Recently, VDR Fokl polymorphism has been associated with decreased Vit D serum concentrations in OSAS patients [46].

A further issue to consider is whether these associations are stronger in patients with DM and OSAS. Such patients were excluded from the present study. The role of Vit D deficiency and the potential utility of its supplementation in type 2 DM remain attractive [47]. In view of the associations herein reported and the important contribution of IR to the development of type two DM [48], it is conceivable that patients with OSAS and type 2 DM would exhibit the strongest association of Vit D deficiency with IR. Should that, indeed, be the case, one might, perhaps, define a clearer indication for Vit D supplementation (type 2 DM with marked IR and OSAS), which has so far proven unreliably efficacious in type 2 DM [47].

## 5. Conclusions

In conclusion, in a cohort of patients diagnosed with OSAS, Vit D levels are decreased in those who have concomitant IR. Obesity may play a role in this process. Further studies are necessary to elucidate the mechanisms underlying the relationship between Vit D and IR in OSAS and to evaluate the role of obesity. Finally, the potential effect of Vit D supplementation in ameliorating IR in such patients merits future evaluation.

## Figures and Tables

**Table 1:** Comparison of anthropometric characteristics between obstructive sleep apnea syndrome (OSAS) patients with (homeostatic model assessment of insulin resistance (HOMA-IR) ≥ 2) and without (HOMA-IR < 2) insulin resistance.

	OSAS Patients With HOMA IR < 2 *n* = 34	OSAS Patients With HOMA IR ≥ 2 *n* = 58	p
**Gender (Male/Female)**	29/5	52/6	0.534
**Age (Years)**	47.7 ± 11.2	51.5 ± 11.9	0.128
**BMI (kg/m^2^)**	32 ± 5.6	36.3 ± 5.8	0.001
**Neck Circumference (cm)**	43.2 ± 3.2	44.6 ± 4	0.104
**Waist Circumference (cm)**	115.1 ± 12.3	122.1 ± 19.1	0.088
**Hip Circumference (cm)**	117.2 ± 13.6	121.2 ± 13.3	0.219
**WHR**	0.98 (0.91–1.05)	1.04 (0.99–1.07)	0.087
**Smoking (%)**	38.2	36.2	0.846

BMI: Body mass index, WHR: Waist to hip ratio.

**Table 2:** Comparison of sleep characteristics between obstructive sleep apnea syndrome (OSAS) patients with (HOMA-IR ≥ 2) and without (HOMA-IR < 2) insulin resistance.

	OSAS Patients With HOMA IR < 2 *n* = 34	OSAS Patients With HOMA IR ≥ 2 *n* = 58	p
**TST (min)**	330 (297.8–350.5)	320 (275.9–350)	0.414
**N1 (%)**	9.2 (2.8–21.5)	11.2 (6.6–18.5)	0.824
**N2 (%)**	67.2 (54.2–74.8)	71.7 (60.2–78.8)	0.103
**N3 (%)**	11.6 (3.3–17.3)	1 (0–10)	0.008
**REM (%)**	10.1 ± 7.8	9 ± 6	0.480
**AHI (events/h)**	40.9 ± 27.9	57.4 ± 28.9	0.009
**Aver SpO_2_ (%)**	92 (88.5–93)	89 (87–91.8)	0.017
**Min SpO_2_ (%)**	74.5 ± 13.9	68 ± 11.5	0.018
**T < 90% (%)**	22.7 (5.4–43.8)	45.1 (22.3–64.9)	0.050
**Arousal Index**	31.5 (13.9–79.1)	44.6 (34.6–66.7)	0.155
**Sleep Efficiency (%)**	87.7 (80.1–91.8)	87.2 (76–93.4)	0.293
**ESS Score**	10 (6–12.5)	10 (8–16)	0.326

AHI: Apnoea hypopnoea index, Aver SpO_2_: Average oxyhaemoglobin saturation, ESS: Epworth sleepiness scale, Min SpO_2_: Minimum oxyhaemoglobin saturation, N1: Sleep stage 1, N2: Sleep stage 2, N3: Sleep stage 3, REM: Rapid eye movement, TST: Total sleep time, T < 90%: Time with oxyhaemoglobin saturation <90%.

**Table 3:** Comparison of laboratory results between obstructive sleep apnea syndrome (OSAS) patients with (HOMA-IR ≥ 2) and without (HOMA-IR < 2) insulin resistance.

	OSAS Patients With HOMA IR < 2 *n* = 34	OSAS Patients With HOMA IR ≥ 2 *n* = 58	p
**FEV_1_ (% predicted)**	96.5 ± 21.3	90.1 ± 21	0.201
**FVC (% predicted)**	95 ± 16.4	85.8 ± 20.9	0.052
**FEV_1_/FVC (%)**	83.2 (78–88.6)	82 (73.2–96.8)	0.485
**pO_2_ (mmHg)**	81.4 ± 12.7	75.3 ± 11.1	0.022
**pCO_2_ (mmHg)**	40 (39–42.4)	40.7 (38.6–44.8)	0.809
**Glucose (mg/dL)**	84 ± 11	104 ± 17	< 0.001
**HOMA–IR**	1.1 (0.5–1.6)	5.5 (3.2–15.1)	< 0.001
**Cholesterol (mg/dL)**	207 ± 48	208 ± 39	0.952
**Triglycerides (mg/dL)**	108 (84–177)	167 (115–227)	0.010
**LDL-C (mg/dL)**	107 (80–148)	109 (100–150)	0.338
**HDL-C (mg/dL)**	48 ± 11	46 ± 13	0.405
**CRP (mg/dL)**	0.25 (0.09–0.63)	0.46 (0.14–0.96)	0.199
**25(OH)D (ng/mL)**	26.7 ± 12.2	19.3 ± 11.5	0.005

CRP: C-reactive protein, FEV_1_: Forced expiratory volume in 1st sec, FVC: Forced vital capacity, HDL-C: High density lipoprotein-cholesterol, HOMA-IR: Homeostatic Model Assessment of Insulin Resistance, LDL-C: Low density lipoprotein-cholesterol, pCO_2_: Carbon dioxide partial pressure, pO_2_: Oxygen partial pressure, 25(OH)D: 25-hydroxyvitamin D.
